# Signal Transducer and Activator of Transcription 3 Hyperactivation Associates With Follicular Helper T Cell Differentiation and Disease Activity in Rheumatoid Arthritis

**DOI:** 10.3389/fimmu.2018.01226

**Published:** 2018-06-04

**Authors:** Jun Deng, Chaofan Fan, Xin Gao, Qunxiong Zeng, Ruru Guo, Yunbo Wei, Zhian Chen, Yanan Chen, Dongcheng Gong, Jia Feng, Yan Xia, Shifei Xiang, Shushi Gong, Lin Yuan, Wei Shen, Wenyan Shen, Lin Lin, Ting Jiang, Dongyi He, Liangjing Lu, Xiaoxiang Chen, Di Yu

**Affiliations:** ^1^China-Australia Centre for Personalised Immunology, Renji Hospital, Shanghai Jiao Tong University School of Medicine, Shanghai, China; ^2^Department of Rheumatology, Shanghai Institute of Rheumatology, Renji Hospital, Shanghai Jiao Tong University School of Medicine, Shanghai, China; ^3^Hubei Provincial Key Laboratory of Occurrence and Intervention of Rheumatic Diseases, Affiliated Hospital of Hubei University for Nationalities, Enshi, China; ^4^Department of Immunology and Infectious Disease, John Curtin School of Medical Research, The Australian National University, Canberra, ACT, Australia; ^5^Laboratory of Immunology for Environment and Health, Shandong Analysis and Test Center, Qilu University of Technology, Shandong Academy of Sciences, Jinan, China; ^6^Department of Rheumatology, Affiliated Hospital of Hubei University for Nationalities, Enshi, China; ^7^Department of Laboratory Medicine, Renji Hospital, Shanghai Jiao Tong University School of Medicine, Shanghai, China; ^8^Department of Laboratory Medicine, Ruijin Hospital, Shanghai Jiao Tong University School of Medicine, Shanghai, China; ^9^Guanghua Hospital of Integrative Chinese and Western Medicine, Shanghai, China

**Keywords:** rheumatoid arthritis, patient, follicular helper T cells, signal transducer and activator of transcription 3, phosphorylation, IL-6

## Abstract

Follicular helper T (Tfh) cells are the specialized CD4^+^ T cell subset that supports B cells to produce high-affinity antibodies and generate humoral memory. Not only is the function of Tfh cells instrumental to mount protect antibodies but also to support autoantibody production and promote systemic inflammation in autoimmune diseases. However, it remains unclear how the activation of Tfh cells is driven in autoimmune diseases. Here, we report that in patients with rheumatoid arthritis (RA), excessive generation of CXCR5^+^PD-1^+^ memory Tfh cells was observed and the frequency of memory Tfh cells correlated with disease activity score calculator for RA (DAS28). The differentiation of Tfh cells is dependent on signal transducer and activator of transcription 3 (STAT3), the key transcription factor downstream of cytokine signal pathways. A drastic increase of phosphorylated STAT3 (pSTAT3) in CD4^+^ T cells were detected in RA patients who also produced larger amounts of STAT3-stimulating cytokines, including IL-6, IL-21, IL-10, and leptin than those of healthy controls. Importantly, the phosphorylation status of STAT3 in CD4^+^ T cells positively correlated with the plasma concentration of IL-6 and the frequency of memory Tfh cells. This study reveals an IL-6-pSTAT3-Tfh immunoregulatory axis in the pathogenesis of RA and reinforces its candidature as biomarkers and targets for diagnosis and therapy.

## Introduction

Rheumatoid arthritis (RA) is a chronic and immune-mediated arthritis that affects up to 1% of the population. It is characterized by synovial inflammation and hyperplasia as well as cartilage and bone destruction (1). Although the name “arthritis” speaks for itself that local tissue remodeling and damage represent the major pathology of RA, the systemic activation of the immune system is instrumental for the disease pathogenesis (1–3). Prior to the onset of clinical disease, increased generation of effector CD4^+^ T cells, elevated production of pro-inflammatory cytokines, and the presence of anticitrullinated protein antibodies (ACPAs) can usually be detected in blood, therefore, being termed as the “pre-rheumatoid arthritis” phase (4).

Follicular helper T (Tfh) cells are a functional CD4^+^ T cell subset that is specialized to support B cells to generate long-lived plasma cells and memory B cells and to produce high-affinity antibodies. By upregulating the chemokine receptor CXCR5, Tfh cells selectively migrate into B-cell follicles and help B cells through the expression of costimulatory receptors including CD40L and OX40 and the secretion of cytokines such as IL-21 (5). The critical role of excessive Tfh cells in the induction of systemic inflammation and the development of autoimmune diseases has been proven by many mouse models (5–8). For RA, there is also accumulated evidence to support a significant contribution of Tfh cells to the development of RA. First, the dependence of Tfh cells has been extensively studied in mouse models. In the commonly used collagen-induced arthritis model, upon the immunization of collagen, CD4^+^ T cells differentiate into Tfh cells to initiate the pathogenic anti-collagen antibody responses (9, 10). In the K/BxN autoimmune arthritis model [KRN T cell receptor transgenic mice on the C57BL/6 (B6) background × non-obese diabetic mice], KRN T cells recognize self-antigen glucose-6-phosphate isomerase (GPI) and differentiate into Tfh cells to promote B cells to produce anti-GPI autoantibodies (11–14). Importantly, blocking of Tfh cell generation was able to prevent the development of diseases in these mouse models. Second, there is a strong involvement of B cells in human RA pathogenesis. The production of high-affinity IgG and IgA antibodies against citrullinated, carbamylated, and acetylated proteins is likely also dependent on Tfh cells (2). Third, several studies including ours have reported excessive Tfh differentiation and function in RA patients as compared to healthy controls (15–19). The association between increased circulating Tfh cells and the presence of high-titer ACPAs and disease activity again suggested a pathogenic role of Tfh cells in RA. The pressing question that remains to be unanswered is the causation of the aberrant Tfh activation in RA.

The development of Tfh cells is driven by signallings *via* T cell receptors through sustained antigen stimulation and co-receptors including CD28 and inducible T-cell costimulator (ICOS). The cytokine milieu also shapes the Tfh differentiation. IL-6 and IL-21 induce the activation of signal transducer and activator of transcription 3 (STAT3) and promote the differentiation of mouse Tfh cells (5). For human Tfh cells, additional cytokines including STAT4-stimulating IL-12 and SMAD2/3-stimulating TGFβ and Activin A were reported to also contribute (5, 20, 21). The activation of STAT3 is essential as the capability of Tfh differentiation was greatly impaired in STAT3-deficient mice (22) and patients carrying functional STAT3 deficiency (23). STAT3 has a strong implication in autoimmune diseases. Monogenic activating *STAT3* mutations were identified in individuals with a spectrum of early-onset autoimmune disease including juvenile-onset arthritis (24). The involvement of the activation of STAT3 and RA was also supported by studies showing enhanced expression of phosphorylated STAT3 (pSTAT3) and STAT3-inducible gene signature in RA patients (25–28). We, therefore, speculated that the hyperactivation of the STAT3 signaling may lead to abnormal Tfh differentiation in RA patients.

In this study, we examined and confirmed the excessive Tfh function in RA patients, shown by the increased frequency of circulating memory Tfh cells and its correlations with disease activity. We found a drastic enhancement of STAT3 phosphorylation in CD4^+^ T cells in RA patients and the activation status of STAT3 positively correlated with the generation of Tfh cells. Major STAT3-stimulating cytokines including IL-6, IL-21, IL-10, and leptin were increased in RA patients and thus contributed to the STAT3 hyperactivation.

## Results

### Characteristics of the Study Subjects

The demographic characteristics of healthy individual controls and patients with RA are shown in Table 1. Thirty-one RA patients including 25 females and 6 males participated the study. Their median age was 60 years. The disease activities of the patient cohort ranged from remission (the disease activity score-28, DAS28 < 2.6) to high (DAS28 > 5.1), with the median value of 4.92. Four naïve patients had no treatment history. The rest patients were treated with low dose of glucocorticoids and/or disease-modifying anti-rheumatic drugs. Those with a treatment history with high-dose glucocorticoids (>10 mg/day) or biologics within the past 6 months were excluded from the study.

**Table 1:** Demographics and clinical data of the study cohorts.

Characteristic	Value
Healthy controls	
Female/male, *n*	22/8
Age, median (range)	55 (18–73)
**Patients with rheumatoid arthritis**	
Female/male, *n*	25/6
Age, median (range)	60 (22–83)
Disease duration (year), median (range)	1 (0–10)
RF, *n* (%)	25 (81%)
Anti-CCP, *n* (%)	26 (84%)
DAS28, median (range)	4.92 (1.27–7.88)
Remission (<2.6), *n* (%)	4 (13%)
Low activity (2.6–3.2), *n* (%)	1 (3%)
Medium activity (3.2–5.1), *n* (%)	11 (35%)
High activity (>5.1), *n* (%)	15 (48%)
CRP (mg/L), median (range)	4.0 (<0.5–106.2)
ESR (mm/h), median (range)	29 (4–84)
WBC (×10^9^/L)	6.5 (3.5–12.0)
Medication, *n* (%)	
Glucocorticoids	18 (58%)
DMARDs	24 (77%)

*RF, rheumatoid factor; Anti-CCP, anti-cyclic citrullinated peptide; DAS28, disease activity score 28; CRP, C-reactive protein; ESR, erythrocyte sedimentation rate; WBC, white blood count; DMARDs, disease-modifying anti-rheumatic drugs*.

### Increased Tfh Cell Differentiation in RA Patients Correlated Disease Activity

**Figure 1:**
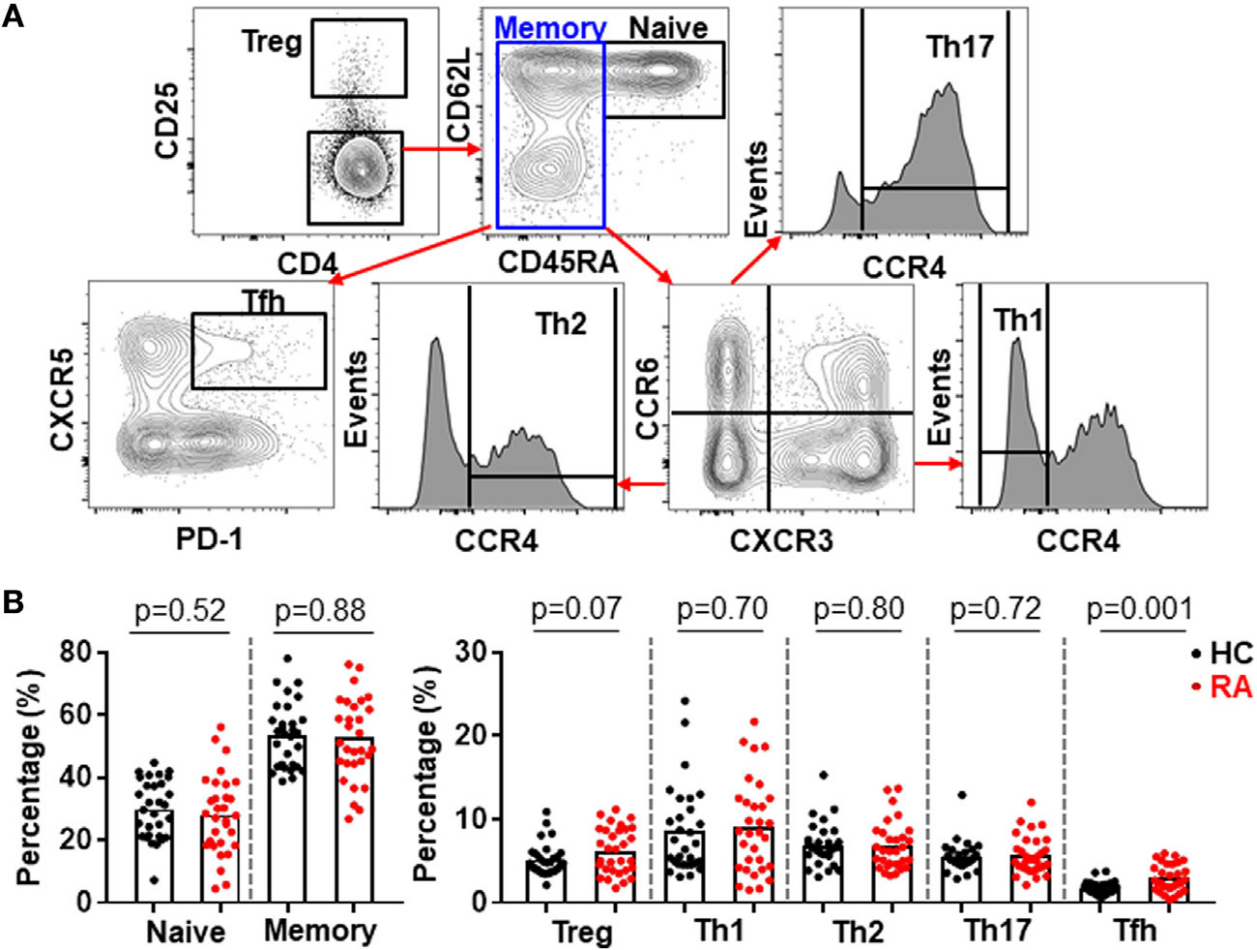
Increased follicular helper T (Tfh) cell differentiation in patients with rheumatoid arthritis (RA). Peripheral blood mononuclear cells from RA patients and healthy control individuals (HC) were analyzed by flow cytometry. **(A)** FACS plots showing the gating of ZA^−^ TCRab^+^ CD4^+^ viable CD4^+^ T cells for Treg (CD25^high^) and conventional CD4^+^ T cell subsets: naïve (CD25^−^CD45RA^+^CD62L^+^), Th1 (CD25^−^CD45RA^−^CXCR3^+^CCR6^−^CCR4^−^), Th2 (CD25^−^CD45RA^−^CXCR3^−^CCR6^−^CCR4^+^), Th17 (CD25^−^CD45RA^−^CXCR3^−^CCR6^+^CCR4^+^), and Tfh (CD25^−^CD45RA^−^CXCR5^+^PD-1^+^). **(B)** Statistics showing the percentages of CD4^+^ T cell subsets in total CD4^+^ T cells. Each dot represents the value of an individual subject with columns showing the mean values of each group. The *p*-values were obtained using Student’s *t*-tests.

**Figure 2:**
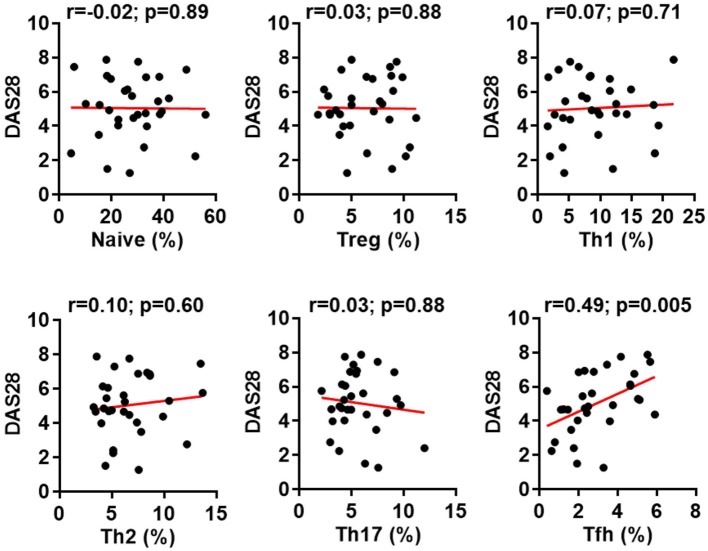
Increased follicular helper T (Tfh) cell differentiation correlates with rheumatoid arthritis (RA) disease activity. The percentages of CD4^+^ T cell subsets in the peripheral blood mononuclear cells from patients with RA were analyzed as Figure 1. The correlation between the frequencies of these subsets and the disease activities measured by DAS28 were determined using Spearman’s correlation coefficient.

CD4^+^ T cells play an important role in the pathogenesis of RA (1–3, 29) but very few efforts have investigated all major CD4^+^ T cell subsets in a single study. Using multicolor flow cytometry, the frequencies of Treg (CD25^high^) and conventional CD4^+^ subsets including naïve (CD25^−^CD45RA^+^CD62L^+^), Th1 (CD25^−^CD45RA^−^CXCR3^+^CCR6^−^CCR4^−^), Th2 (CD25^−^CD45RA^−^CXCR3^−^CCR6^−^CCR4^+^), Th17 (CD25^−^CD45RA^−^CXCR3^−^CCR6^+^CCR4^+^), and Tfh (CD25^−^CD45RA^−^CXCR5^+^PD-1^+^) subsets in CD4^+^ T cells were analyzed simultaneously (30) (Figure 1A; Figure S1 in Supplementary Material). The comparison between these subsets in healthy individuals and RA patients revealed a significantly higher frequency of Tfh cells in RA patients, increasing from an average of 1.6% in the controls to 2.6% in RA patients (Figure 1B). On the contrary, we did not observe any significant difference for other CD4^+^ T cell subsets between healthy individuals and RA patients. We also examined the potential correlations between the frequencies of CD4^+^ T cell subsets and disease activities as measured by DAS28. Again, only Tfh cells’ frequency, but not others’ showed a positive correlation with DAS28 values (*p*-value = 0.005) (Figure 2). The increase of the Tfh activation and the correlation between the aberrant Tfh differentiation and RA disease activity support the notion that Tfh cells participate in the pathogenesis of RA.

### Hyperactivation of STAT3 in CD4^+^ T Cells Correlated With Tfh Differentiation in RA Patients

**Figure 3:**
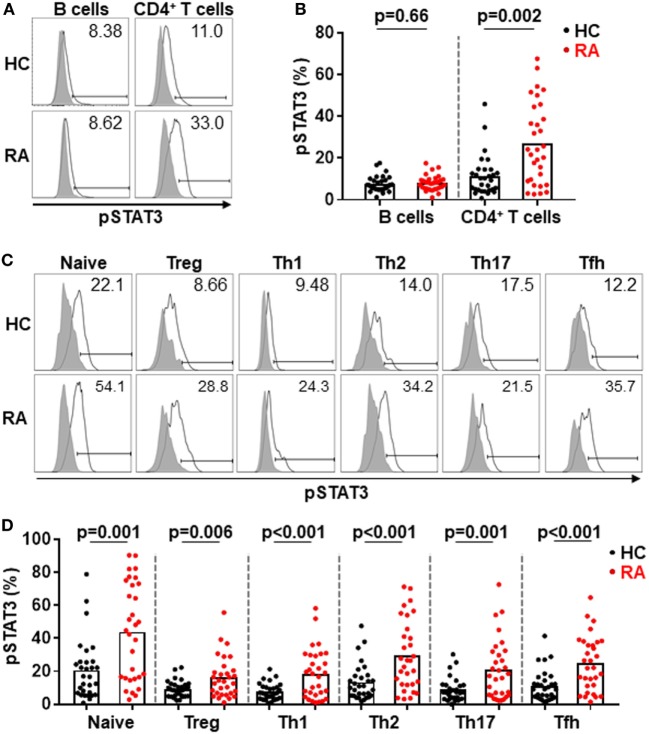
Constitutive phosphorylation of signal transducer and activator of transcription 3 (STAT3) in CD4^+^ T cells from patients with rheumatoid arthritis (RA). The expression of intracellular phosphorylated STAT3 (pSTAT3) was analyzed using Phosflow assays in CD4^+^ T cells and B cells from RA patients. **(A)** FACS plots showing representative staining patterns for pSTAT3 (empty histograms) and an isotype control antibody (filled histograms) of indicated immune cell types; Numbers indicating the percentages of the pSTAT3 positive population. **(B)** Statistics showing the percentages of pSTAT3 positive population in total ZA^−^CD3^−^CD19^+^ viable B cells and ZA^−^TCRab^+^CD4^+^ viable CD4^+^ T cells from RA or HC groups. **(C)** FACS plots showing representative staining patterns for pSTAT3 and an isotype control antibody of indicated CD4^+^ T cell subsets; numbers indicating the percentages of the pSTAT3 positive population. **(D)** Statistics showing the percentages of pSTAT3 positive population in indicated CD4^+^ T cell subsets from RA or HC groups. Each dot represents the value of an individual subject with columns showing the mean value of each group. The *p*-values were obtained using Student’s *t*-tests.

The increase of Tfh cells in RA was reported in previous studies (15–19) and confirmed in our cohort. However, the reason that caused such immune dysregulation remained unknown. We hypothesized the constitutive activation of STAT3 in RA (25–28) could promote the Tfh differentiation since STAT3 is pivotal for the generation of Tfh cells (5). We took the advantage of Phosflow to quantify the phosphorylation of STAT3 in CD4^+^ T cells. We could detect substantial expression of pSTAT3 in CD4^+^ T cells but not in B cells (Figure 3A). Importantly, the expression of pSTAT3 in CD4^+^ T cells from RA patients (mean value: 27.0%) was 2.4-fold higher than that of CD4^+^ T cells from healthy controls (mean value: 11.4%) (*p* = 0.002) (Figure 3B). We did not detect any difference of pSTAT3 expression in B cells between the two groups. We further analyzed the expression on pSTAT3 in each CD4^+^ T cell subset as shown in Figure 1A. To our surprise, all CD4^+^ T cell subsets had significantly higher level of pSTAT3 in RA patients than those in HC (Figure 3C). There was an approximate twofold increase of pSTAT3 in each of CD4^+^ T cell subsets in RA as compared to those in healthy controls (Figure 3D).

**Figure 4:**
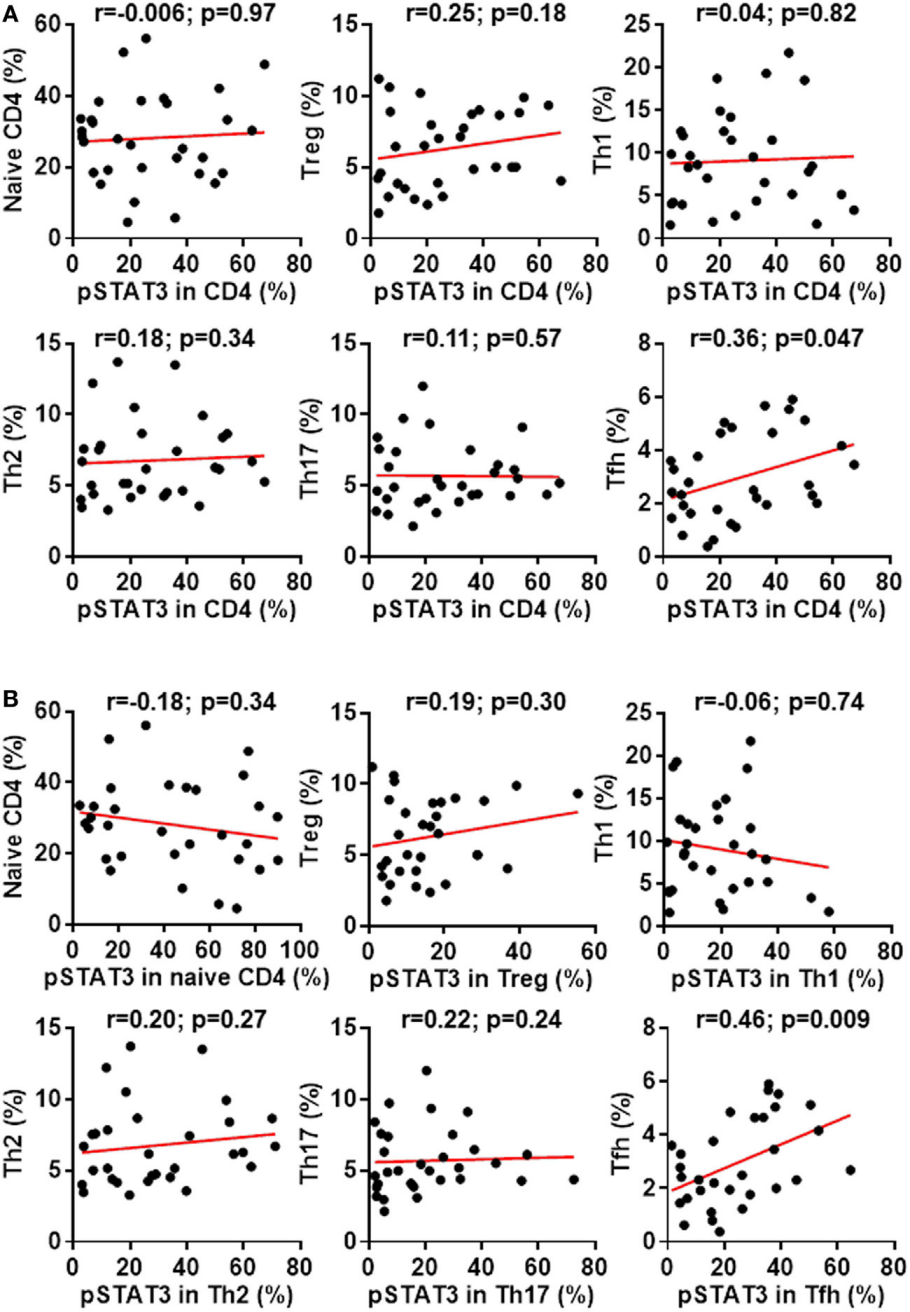
Signal transducer and activator of transcription 3 (STAT3) hyperactivation correlates with aberrant follicular helper T (Tfh) differentiation in patients with rheumatoid arthritis (RA). Statistics showing the relationship between the frequencies of indicated CD4^+^ T cell subsets with the phosphorylated STAT3 (pSTAT3) expression in total CD4^+^ T cells **(A)** or in each individual subsets **(B)** in the peripheral blood mononuclear cells from patients with RA. The correlation was determined using Spearman’s correlation coefficient.

We then asked whether the phosphorylation of STAT3 associated with the generation of Tfh cells or other CD4^+^ T cell subsets in RA. By analyzing the relationship between the pSTAT3 expression in total CD4^+^ T cells and the frequencies of CD4^+^ T cell subsets, we could only detect a modest but significant correlation between the status of STAT3 phosphorylation in CD4^+^ T cells and the Tfh generation (*p*-value = 0.047) (Figure 4A). Intriguingly, we also detected a specific correlation of between STAT3 phosphorylation in CD4^+^ T cells and Tfh frequencies in HC (Figure S2 in Supplementary Material). It was possible that the differentiation of each CD4^+^ T cells might be more specifically affected by the phosphorylation of STAT3 in their own. To test this, we performed a similar study to examine the relationship between the pSTAT3 expression in individual CD4^+^ T cell subset and their frequencies. The STAT3 phosphorylation showed no correlation with the frequencies of naïve, Treg, Th1, Th2, or Th17 cells. The correlation with Tfh cells, nevertheless, became more obvious (*p*-value = 0.009) (Figure 4B). The results strongly suggest the hyperactivated STAT3 drove the Tfh differentiation in patients with RA.

**Figure 5:**
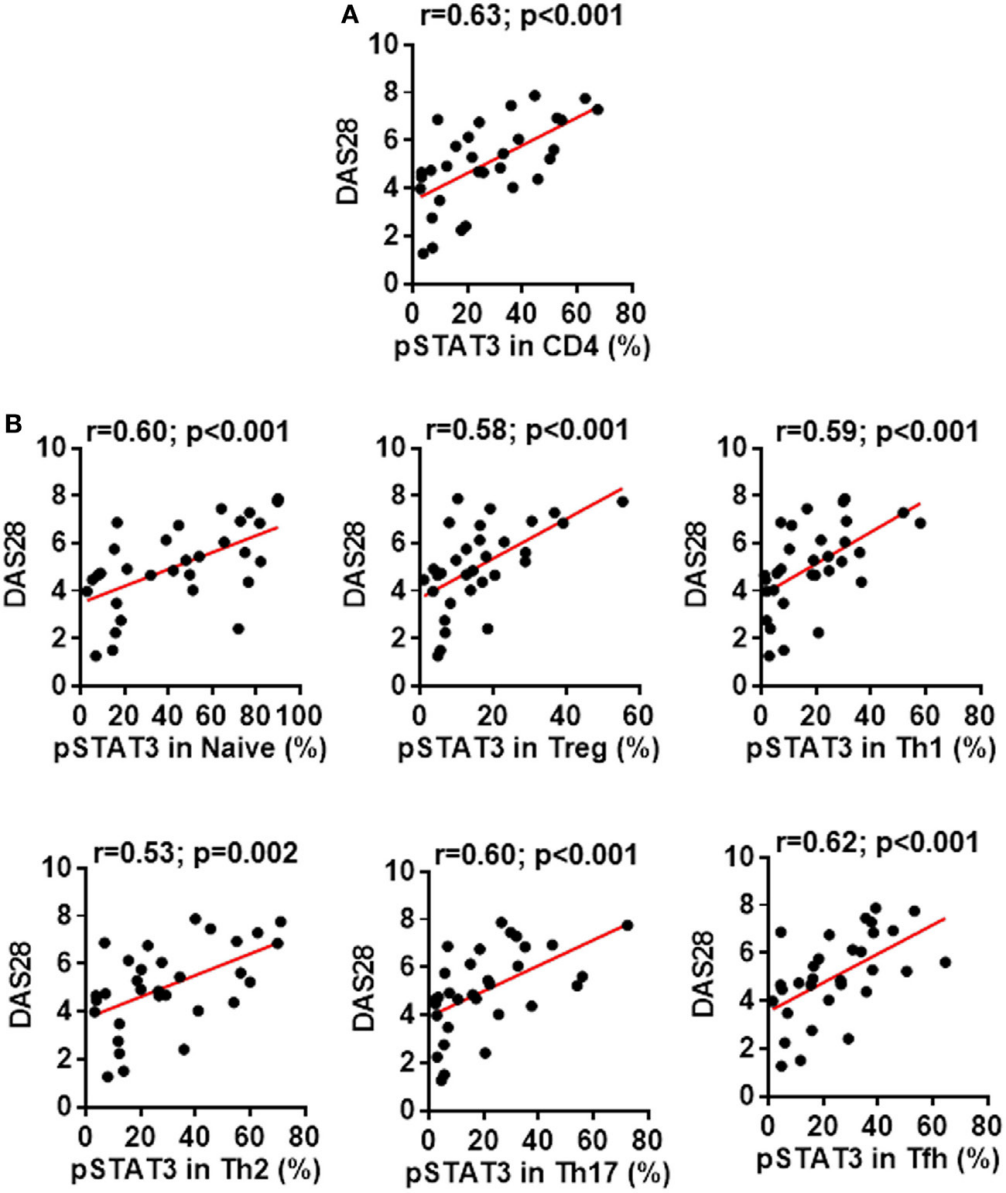
Signal transducer and activator of transcription 3 (STAT3) hyperactivation correlates with rheumatoid arthritis (RA) disease activity. Statistics showing the relationship between the disease activities measured by DAS28 with the phosphorylated STAT3 (pSTAT3) expression in total CD4^+^ T cells **(A)** or in each individual subsets **(B)** in the peripheral blood mononuclear cells from patients with RA. The correlation was determined using Spearman’s correlation coefficient.

In addition to dissecting a role of the STAT3 pathway in regulating CD4^+^ T cell differentiation, we also tested whether the status of STAT3 phosphorylation might affect the disease severity. We found a clear correlation between patients’ disease activities and the pSTAT3 expression, not only in total CD4^+^ T cells (Figure 5A) but also in each CD4^+^ T cell subsets (Figure 5B). This indicates the contribution of STAT3 hyperactivation to RA is not limited to a specific CD4^+^ T cell population, but rather broad.

### STAT3-Stimulating Cytokines Were Elevated in RA Patients

**Figure 6:**
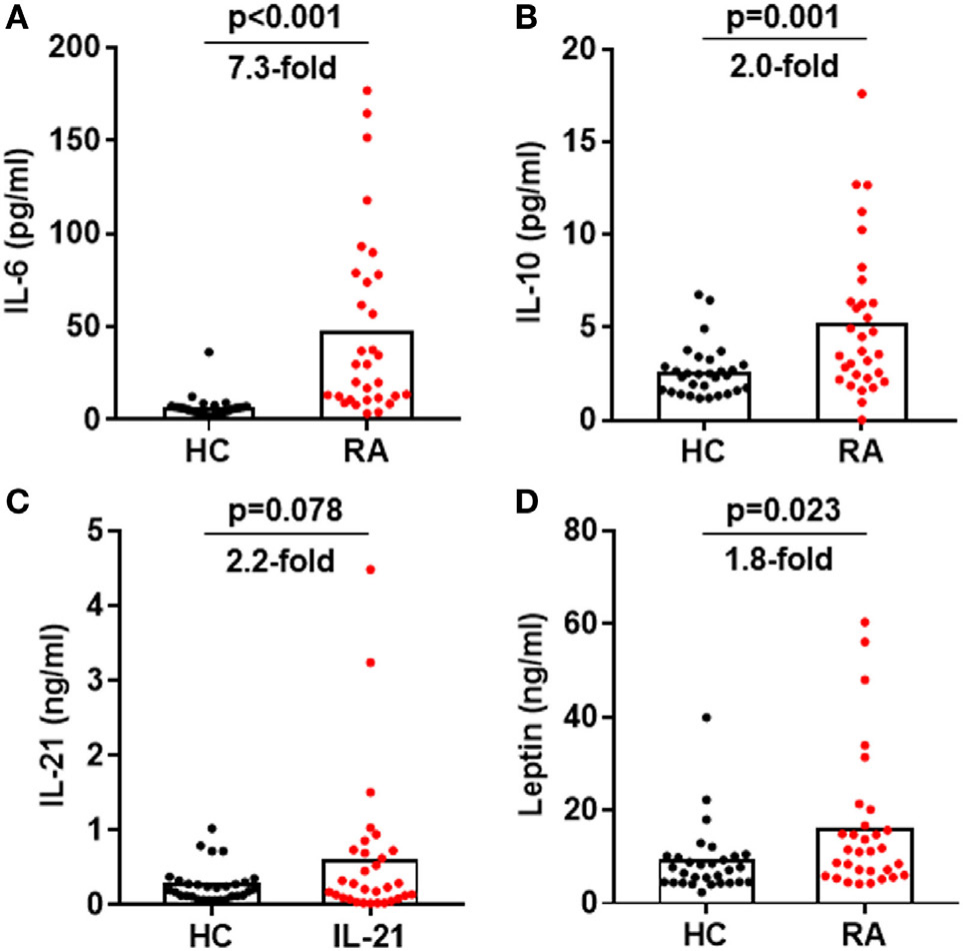
Increased signal transducer and activator of transcription 3-stimulating cytokines in the plasma of rheumatoid arthritis (RA) patients. Plasma was isolated from blood from RA patients and healthy controls. The amount of IL-6 **(A)**, IL-10 **(B)**, IL-21 **(C)**, and leptin **(D)** was measured by ELISA. Each dot represents the value of an individual subject with columns showing the mean values of each group. The *p*-values were obtained using Student’ *t*-tests.

**Figure 7:**
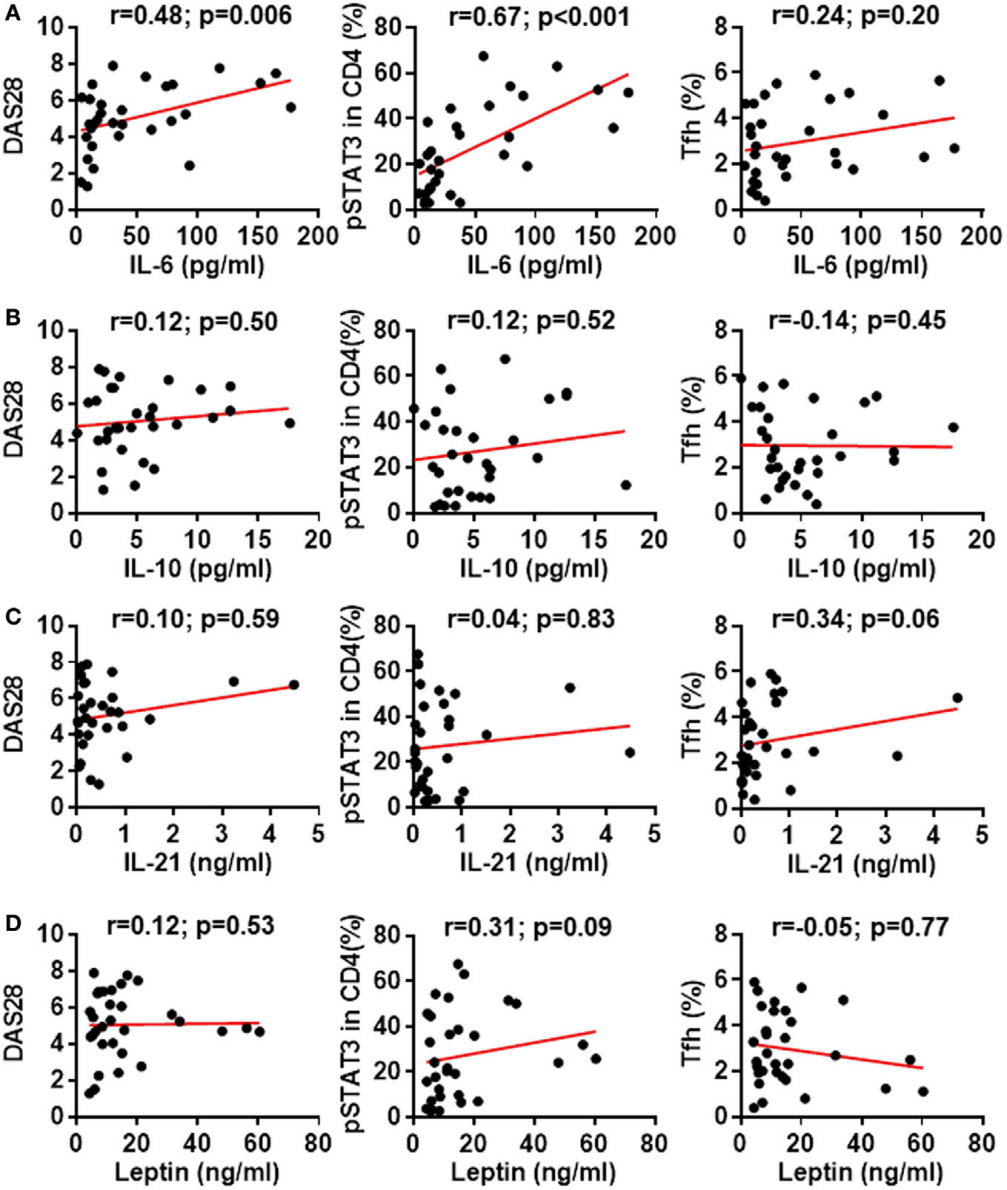
Association between signal transducer and activator of transcription 3 (STAT3)-stimulating cytokines with STAT3 phosphorylation, disease activity or follicular helper T (Tfh) differentiation. Statistics showing the relationship between the expression of phosphorylated STAT3 (pSTAT3) in total CD4^+^ T cells, the disease activities measured by DAS28 or the frequency of Tfh cells with the amount of plasma IL-6 **(A)**, IL-10 **(B)**, IL-21 **(C)**, or leptin **(D)**. The correlation was determined using Spearman’s correlation coefficient.

Cytokines IL-6, IL-10, IL-21, and leptin can activate STAT3 phosphorylation in CD4^+^ T cells (31, 32). Could these cytokines induce the enhanced STAT3 phosphorylation in RA patients? We first measured the production of these cytokines in RA patients’ plasma. Results by ELISA demonstrated upregulated production of all four cytokines with the most prominent increase for IL-6: a 7.3-fold upregulation from 6.6 ± 1.1 pg/mL in healthy individuals to 48.06 ± 8.9 pg/mL in RA patients (Figure 6A). The increase for IL-10, IL-21, and leptin was about twofold (Figures 6B–D). To define the contribution of the elevated pro-inflammatory cytokines to the hyperactivation of STAT3 and the disease activities, we analyzed the relationship between cytokine levels and the disease activity DAS28 or the pSTAT3 expression. IL-6 levels significantly correlated with DAS28 (*p*-value = 0.006) and pSTAT3 (*p*-value < 0.001) (Figure 7A). Other cytokines also showed general trends for positive correlations but not significant (Figures 7B–D). These results spotlight a central role of IL-6 in inducing pSTAT3 in CD4^+^ T cells and for the development of the disease. We also test whether there was a relationship between the Tfh frequency and any of these STAT3-stimulating cytokines but could not identify any substantial link. We thus conclude that although the Tfh differentiation in RA patients is critically driven by the STAT3 phosphorylation, it may not depend on a single cytokine. A synergistic effect of multiple cytokines or other signals are candidate mechanisms.

## Discussion

CD4^+^ T cells have long been regarded as a key player in the pathogenesis of RA (29). Indeed, genetic variants in the major histocompatibility complex (MHC) region, especially MHC class II *HLA-DRB1* genes contribute to the overall 11–37% genetic risk of RA. Non-HLA genes encoding molecules directly involved in pathways of T-cell function including *PTPN22, IL23R, CTLA4, STAT4*, and *CD40* are also ranked high in the identified RA-risk loci (33). Although early studies discovered synovial effector CD4^+^ T cells predominantly produce Th1 cytokines IFNγ and TNFα (34), more recent research has been focusing on later discovered CD4^+^ T cell subsets: Th17 and Tfh cells.

In the cohort of RA patients we examined, there was a significant increase of CXCR5^+^PD-1^+^ memory Tfh cells in blood, as compared to that of healthy controls. The frequency of Tfh cells positively correlated with the disease activities, as measured by DAS28. This observation was largely in agreement with several published reports showing the aberrant function of Tfh cells in RA patients (15–19). The circulating memory Tfh cells can be analyzed in two different ways (35, 36). Studies using CXCR5^+^PD-1^+^ or CXCR5^+^ICOS^+^ to mark Tfh cells indicated an active Tfh differentiation in RA (15, 16, 18) while those applying CCR6 and CCR3 to stratify Tfh cells demonstrated a biased Tfh polarization into CXCR5^+^CCR6^+^CXCR3^−^ (Th17-type Tfh) and/or CXCR5^+^CCR6^−^CXCR3^−^ (Th2-type Tfh) subsets in RA patients (18, 19). Despite different analytic methodologies, such evidence strongly supports a significant contribution of Tfh cells to systemic inflammation and autoimmunity that drive the development of RA. To be noted, the phenotype of circulating Tfh cells is different from B-helper T cells infiltrated in inflammatory synovial tissues in RA. The latter CD4^+^ T cell population, though expressing conventional Tfh markers including PD-1, ICOS, CXCL13, and IL-21, do not upregulate CXCR5 (37). It remains unclear whether these B-helper T cells in joints also participate in systemic immune activation in RA.

There is a major question left unanswered by the studies that characterized Tfh cells in RA patients—what induces the aberrant Tfh differentiation? We set to test the hypothesis that the constitutive activation of STAT3 in RA led to enhanced Tfh differentiation. By measuring the phosphorylation of STAT3, we found a drastic increase of pSTAT3 in CD4^+^ T cells but not in B cells. The increase of pSTAT3 was detected in all CD4^+^ T cell subsets with highest phosphorylation status in naïve CD4^+^ T cells. In human CD4^+^ T cells, naïve CD4^+^ T cells were shown to express a lower level of suppressor of cytokine signaling 3 (SOCS3), which interacts with cytokine receptors and inhibits receptor-mediated signal transduction (38). As the induction of SOCS3 represents the key mechanism to block the activation of STAT3 (39), the low expression of SOCS3 may lead to a high pSTAT3 in naïve CD4^+^ T cells. For effector CD4^+^ T cell subsets, pSTAT3 was detected lowest in Th1 cells, reflecting the fact that STAT3 functions to promote Th2, Th17, and Tfh differentiation but inhibit Th1 differentiation (22, 23, 39–43). Despite the high levels of pSTAT3 in all three subsets, the intensity of pSTAT3 in CD4^+^ T cells only correlated with the frequency of Tfh cells but not Th2 or Th17 cells, suggesting redundant factors other than STAT3 potently regulate Th2 and Th17 differentiation. The key role of pSTAT3 in promoting the Tfh generation was further recognized by a strong correlation between the frequency of Tfh cells and the pSTAT3 expression in Tfh cells.

Since the STAT3 hyperactivation was observed across all CD4^+^ T cells, we believe it was caused a systemic change of the immune system that is not specific for certain subsets. We measured the serum concentrations of cytokines IL-6, IL-10, IL-21, and leptin, all of which have been shown to induce the phosphorylation of STAT3 in T cells (31, 32). All of these cytokines were upregulated in RA patients as compared to healthy controls, with a prominent increase of IL-6 for more than sevenfold. By analyzing the correlation between the plasma cytokine concentrations and the pSTAT3 status in CD4^+^ T cells, IL-6 appears to play a central role in determining the activation of STAT3 in CD4^+^ T cells. Other cytokines may synergistically enhance pSTAT3 as there were general trends for positive correlations. It has been previously shown that serum IL-21 concentrations modestly correlated with the frequencies of Tfh cells in RA patients (44). In our cohort, we also observed a similar trend of positive correlation between IL-21 concentration and Tfh activation, but it was not statistically significant (*p*-value = 0.06). There were no significant correlation between the frequency of Tfh cells and the concentration of any of tested STAT3-activating cytokines. It could be well explained by the fact that the Tfh differentiation is induced by the combination of multiple cytokines (20). It is also possible that cytokines may preferably execute in supporting Tfh differentiation in local lymphoid tissue spleen and lymph nodes so the plasma concentrations were not always representative of its tissue concentrations.

Our study revealed an IL-6-pSTAT3-Tfh axis in regulating immune responses in RA patients. As the status of each of three components individually correlated with the disease activity indicator DAS 28, this immunoregulatory axis is likely to reside proximal to the center of RA pathogenesis. Therapeutics have been developed to target this axis including monoclonal antibodies to block IL-6 and small molecules to inhibit Janus kinases (JAKs) that act upstream of the STAT3 pathway. IL-6 blockers such as tocilizumab and JAK inhibitors such as tofacitinib have demonstrated profound efficacies and been approved to treat RA (45). Intriguingly, tocilizumab therapy was shown not only reducing pSTAT3 in CD4^+^ T cells (46) but also inhibiting the Tfh differentiation (47). The evidence gained here and those from previous studies justify targeting the IL-6-pSTAT3-Tfh axis for biomarker discovery and drug development for RA diagnosis and therapy.

## Materials and Methods

### Study Subjects

Thirty-one patients diagnosed as RA according to the 2010 RA classification criteria by American College of Rheumatology/European League Against Rheumatism (48) were recruited. The cohort included both newly diagnosed patients (*n* = 4) and patients with long-standing disease (*n* = 27). No patients had a history of treatment with biologic agents or high-dose corticosteroids (>10 mg/day) 6 months preceding the study. Thirty healthy individuals with comparable demographics were recruited as controls. All participants consented in writing to donate blood for the study. Ethics was approved by human ethics committees of Renji Hospital and Ruijin Hospital affiliated to Shanghai Jiao Tong University School of Medicine (Shanghai, China) and Affiliated Hospital of Hubei University for Nationalities (Hubei, China).

### Isolation of Peripheral Blood Mononuclear Cells (PBMCs) and Plasma

Blood from healthy individuals and patients with RA were collected in BD Vacutainer^®^ Blood Collection Tubes (BD). After the centrifugation (300 *g*, 20°C, 5 min), plasma was collected and stored in −80°C for further analysis. Cells were diluted with PBS (1:1), and gently loaded to Ficoll-Paque Plus (GE) layer at the ratio of 1:1 (PBS + blood cells:Ficoll), followed by density gradient centrifugation (450 *g*, 20°C, 20 min, no brake). Mononuclear cell layer was transferred to a new tube and washed with cold PBS. Cells were suspended in cold FACS buffer (1% BSA, 0.05% NaN3) for further analysis.

### Flow Cytometric Analysis

Freshly isolated PBMCs were incubated with following fluorochrome-conjugated monoclonal antibodies in FACS buffer for surface staining. Antibodies (from BD Biosciences and BioLegend) were TCRαβ (clone IP26), CXCR3 (clone G025H7), CD45RA (clone HI100), CD3 (clone UCHT1), PD-1 (clone EH12.2H7), CD19 (clone SJ25C1), CCR6 (clone 11A9), CD8a (clone SK1), CCR4 (clone L291H4), CD62L (clone DREG-56), CD25 (clone BC96), CXCR5 (clone RF8B2), CD4 (clone RPA-T4), and dead cells stained with Zombie Aqua were excluded from analysis. After the surface staining, cells were washed with FACS buffer and then fixed with pre-warmed Phosflow™ Fix Buffer I (BD) in 37°C for 15 min. Cells were washed and resuspended in pre-chilled Phosflow Perm Buffer III (BD) in 4°C for 25 min. After wash, cells were stained with anti-pSTAT3 (BD, clone 4/pSTAT3) at 37°C for 30 min to detect pSTAT3 (pY705). The same procedure was performed to stain with an isotype control antibody (BD, clone G155-178). The expression of surface markers and intracellular pSTAT3 were analyzed by a FACS analyser (LSRFortessa X-20, BD). The results were analyzed with FlowJo software (TreeStar).

### ELISA

Cytokines in plasma samples were measured by ELISA kits (Biolegend: IL-6 and IL-21; Invitrogen: IL-10; R&D systems: leptin) following the manufacturers’ protocol. Plasma was diluted at 1:5 for IL-6, IL-10, and IL-21 and 1:100 for leptin, using ELISA diluent (1% BSA in PBS-T). Standards and diluted-plasma samples were incubated at room temperature for 2 h, followed by incubations with detecting antibodies and streptavidin-horseradish peroxidase. The substrate 3,3′,5,5′-Tetramethylbenzidine was added before the values of optical densities were obtained by a microplate reader (SpectraMax 190, Molecular Devices).

### Quantitative PCR

Naïve (CD25^−^CD45RA^+^CD62L^+^), Th1 (CD25^−^CD45RA^−^CXCR3^+^CCR6^−^CCR4^−^), Th2 (CD25^−^CD45RA^−^CXCR3^−^CCR6^−^CCR4^+^), and Th17 (CD25^−^CD45RA^−^CXCR3^−^CCR6^+^CCR4^+^) cells were sorted with a FACS cell sorter (BD Aria III). mRNA samples were extracted with Trizol reagents, and cDNA were synthesized with cDNA Synthesis Kit (Takara). Following, *Tbx21* forward 5′-ATTGCCGTGACTGCCTACCAGA-3′ and reverse 5′-GGAATTGACAGTTGGGTCCAGG-3′; *Gata3* forward 5′-ACCACAACCACACTCTGGAGGA-3′ and reverse 5′-TCGGTTTCTGGTCTGGATGCCT-3′; *Rorc* forward 5′-GAGGAAGTGACTGGCTACCAGA-3′ and reverse 5′-GCACAATCTGGTCATTCTGGCAG-3′; *Ifng* forward 5′-GAGTGTGGAGACCATCAAGGAAG-3′ and reverse 5′-TGCTTTGCGTTGGACATTCAAGTC-3′; *Il4* forward 5′-CCGTAACAGACATCTTTGCTGCC-3′ and reverse 5′-GAGTGTCCTTCTCATGGTGGCT-3′; *Il17a* forward 5′-CGGACTGTGATGGTCAACCTGA-3′ and reverse 5′-GCACTTTGCCTCCCAGATCACA-3′; *Gapdh* forward 5′-GTCTCCTCTGACTTCAACAGCG-3′ and reverse 5′-ACCACCCTGTTGCTGTAGCCAA-3′, were used to measure the transcript levels using SYBR (Takara) with a Real-Time PCR Systems (QuantStudio 7 Flex, ABI). Relative fold change of gene expression was calculated by 2^−(DCT experiment−DCT control)^. DCT = CT^gene of interest^ − CT^GAPDH^.

### Statistical Analysis

Data were analyzed with GraphPad Prism (version 7.0, GraphPad Software). The correlations of indicated parameters were determined by Spearman’s correlation coefficient. Two-tailed *t*-tests were used to compare parameters between healthy individuals and RA patients. Results were considered statistically significant when *p-*values < 0.05.

## Ethics Statement

Ethics was approved by human ethics committees of Renji Hospital and Ruijin Hospital affiliated to Shanghai Jiao Tong University School of Medicine (Shanghai, China) and Affiliated Hospital of Hubei University for Nationalities (Hubei, China).

## Author Contributions

DY, JD, XC, and LLu conceived the study. DY, JD, and XG designed the experiments. XC, LLu, CF, RG, JF, YX, SX, SG, LY, WeiS, WenyanS, LLin, TJ, and DH helped to recruit patients and collect samples. JD, XG, QZ, DG, ZC, YC, and YW performed experiments and collected data. JD, XG, CF, LLu, XC, and DY interpreted the data and wrote the manuscript. All authors approved the final version.

## Conflict of Interest Statement

The authors declare that the research was conducted in the absence of any commercial or financial relationships that could be construed as a potential conflict of interest.

## References

[1] Smolen JS Aletaha D McInnes IB. Rheumatoid arthritis. Lancet (2016) 388:2023–38. 10.1016/S0140-6736(16)30173-8 27156434

[2] Malmstrom V Catrina AI Klareskog L. The immunopathogenesis of seropositive rheumatoid arthritis: from triggering to targeting. Nat Rev Immunol (2017) 17:60–75. 10.1038/nri.2016.124 27916980

[3] McInnes IB Schett G. Cytokines in the pathogenesis of rheumatoid arthritis. Nat Rev Immunol (2007) 7:429–42. 10.1038/nri2094 17525752

[4] Mankia K Emery P Preclinical rheumatoid arthritis: progress toward prevention. Arthritis Rheumatol (2016) 68:779–88. 10.1002/art.39603 26814677

[5] Vinuesa CG Linterman MA Yu D MacLennan IC. Follicular helper T cells. Annu Rev Immunol (2016) 34:335–68. 10.1146/annurev-immunol-041015-055605 26907215

[6] Linterman MA Rigby RJ Wong RK Yu D Brink R Cannons JL Follicular helper T cells are required for systemic autoimmunity. J Exp Med (2009) 206:561–76. 10.1084/jem.20081886 19221396 PMC2699132

[7] Yu D Vinuesa CG. Multiple checkpoints keep follicular helper T cells under control to prevent autoimmunity. Cell Mol Immunol (2010) 7:198–203. 10.1038/cmi.2010.18 20364160 PMC4002912

[8] Craft JE. Follicular helper T cells in immunity and systemic autoimmunity. Nat Rev Rheumatol (2012) 8:337–47. 10.1038/nrrheum.2012.58 22549246 PMC3604997

[9] Zhong MC Veillette A. The adaptor molecule signaling lymphocytic activation molecule (SLAM)-associated protein (SAP) is essential in mechanisms involving the Fyn tyrosine kinase for induction and progression of collagen-induced arthritis. J Biol Chem (2013) 288:31423–36. 10.1074/jbc.M113.473736 24045941 PMC3814739

[10] Moschovakis GL Bubke A Friedrichsen M Falk CS Feederle R Forster R T cell specific Cxcr5 deficiency prevents rheumatoid arthritis. Sci Rep (2017) 7:8933 10.1038/s41598-017-08935-6 28827539 PMC5567121

[11] Victoratos P Kollias G. Induction of autoantibody-mediated spontaneous arthritis critically depends on follicular dendritic cells. Immunity (2009) 30:130–42. 10.1016/j.immuni.2008.10.019 19119026

[12] Block KE Zheng Z Dent AL Kee BL Huang H. Gut microbiota regulates K/BxN autoimmune arthritis through follicular helper T but not Th17 cells. J Immunol (2016) 196:1550–7. 10.4049/jimmunol.1501904 26783341 PMC4744513

[13] Chevalier N Macia L Tan JK Mason LJ Robert R Thorburn AN The role of follicular helper T cell molecules and environmental influences in autoantibody production and progression to inflammatory arthritis in mice. Arthritis Rheumatol (2016) 68:1026–38. 10.1002/art.39481 26501485

[14] Teng F Klinger CN Felix KM Bradley CP Wu E Tran NL Gut microbiota drive autoimmune arthritis by promoting differentiation and migration of peyer’s patch T follicular helper cells. Immunity (2016) 44:875–88. 10.1016/j.immuni.2016.03.013 27096318 PMC5296410

[15] Ma J Zhu C Ma B Tian J Baidoo SE Mao C Increased frequency of circulating follicular helper T cells in patients with rheumatoid arthritis. Clin Dev Immunol (2012) 2012:827480. 10.1155/2012/827480 22649468 PMC3357937

[16] He J Tsai LM Leong YA Hu X Ma CS Chevalier N Circulating precursor CCR7(lo)PD-1(hi) CXCR5(+) CD4(+) T cells indicate Tfh cell activity and promote antibody responses upon antigen reexposure. Immunity (2013) 39:770–81. 10.1016/j.immuni.2013.09.007 24138884

[17] Wang J Shan Y Jiang Z Feng J Li C Ma L High frequencies of activated B cells and T follicular helper cells are correlated with disease activity in patients with new-onset rheumatoid arthritis. Clin Exp Immunol (2013) 174:212–20. 10.1111/cei.12162 23786438 PMC3828824

[18] Arroyo-Villa I Bautista-Caro MB Balsa A Aguado-Acin P Bonilla-Hernan MG Plasencia C Constitutively altered frequencies of circulating follicullar helper T cell counterparts and their subsets in rheumatoid arthritis. Arthritis Res Ther (2014) 16:500. 10.1186/s13075-014-0500-6 25475240 PMC4275955

[19] Singh D Henkel M Sendon B Feng J Fabio A Metes D Analysis of CXCR5(+)Th17 cells in relation to disease activity and TNF inhibitor therapy in rheumatoid arthritis. Sci Rep (2016) 6:39474 10.1038/srep39474 28004828 PMC5177940

[20] Ueno H Banchereau J Vinuesa CG. Pathophysiology of T follicular helper cells in humans and mice. Nat Immunol (2015) 16:142–52. 10.1038/ni.3054 25594465 PMC4459756

[21] Locci M Wu JE Arumemi F Mikulski Z Dahlberg C Miller AT Activin A programs the differentiation of human TFH cells. Nat Immunol (2016) 17:976–84. 10.1038/ni.3494 27376469 PMC4955732

[22] Ray JP Marshall HD Laidlaw BJ Staron MM Kaech SM Craft J. Transcription factor STAT3 and type I interferons are corepressive insulators for differentiation of follicular helper and T helper 1 cells. Immunity (2014) 40:367–77. 10.1016/j.immuni.2014.02.005 24631156 PMC3992517

[23] Ma CS Avery DT Chan A Batten M Bustamante J Boisson-Dupuis S Functional STAT3 deficiency compromises the generation of human T follicular helper cells. Blood (2012) 119:3997–4008. 10.1182/blood-2011-11-392985 22403255 PMC3355712

[24] Flanagan SE Haapaniemi E Russell MA Caswell R Allen HL De Franco E Activating germline mutations in STAT3 cause early-onset multi-organ autoimmune disease. Nat Genet (2014) 46:812–4. 10.1038/ng.3040 25038750 PMC4129488

[25] Pratt AG Swan DC Richardson S Wilson G Hilkens CM Young DA A CD4 T cell gene signature for early rheumatoid arthritis implicates interleukin 6-mediated STAT3 signalling, particularly in anti-citrullinated peptide antibody-negative disease. Ann Rheum Dis (2012) 71:1374–81. 10.1136/annrheumdis-2011-200968 22532634 PMC3396452

[26] Isomaki P Junttila I Vidqvist KL Korpela M Silvennoinen O. The activity of JAK-STAT pathways in rheumatoid arthritis: constitutive activation of STAT3 correlates with interleukin 6 levels. Rheumatology (Oxford) (2015) 54:1103–13. 10.1093/rheumatology/keu430 25406356

[27] Kuuliala K Kuuliala A Koivuniemi R Oksanen S Hamalainen M Moilanen E Constitutive STAT3 phosphorylation in circulating CD4+ T lymphocytes associates with disease activity and treatment response in recent-onset rheumatoid arthritis. PLoS One (2015) 10:e0137385. 10.1371/journal.pone.0137385 26353115 PMC4564221

[28] Anderson AE Pratt AG Sedhom MA Doran JP Routledge C Hargreaves B IL-6-driven STAT signalling in circulating CD4+ lymphocytes is a marker for early anticitrullinated peptide antibody-negative rheumatoid arthritis. Ann Rheum Dis (2016) 75:466–73. 10.1136/annrheumdis-2014-205850 25649145 PMC4752669

[29] Cope AP T cells in rheumatoid arthritis. Arthritis Res Ther (2008) 10(Suppl 1):S1 10.1186/ar2412 PMC2582813 19007421

[30] He J Zhang X Wei Y Sun X Chen Y Deng J Low-dose interleukin-2 treatment selectively modulates CD4(+) T cell subsets in patients with systemic lupus erythematosus. Nat Med (2016) 22:991–3. 10.1038/nm.4148 27500725

[31] O’Shea JJ Schwartz DM Villarino AV Gadina M McInnes IB Laurence A. The JAK-STAT pathway: impact on human disease and therapeutic intervention. Annu Rev Med (2015) 66:311–28. 10.1146/annurev-med-051113-024537 25587654 PMC5634336

[32] Naylor C Petri WA Jr. Leptin regulation of immune responses. Trends Mol Med (2016) 22:88–98. 10.1016/j.molmed.2015.12.001 26776093

[33] Kim K Bang SY Lee HS Bae SC. Update on the genetic architecture of rheumatoid arthritis. Nat Rev Rheumatol (2017) 13:13–24. 10.1038/nrrheum.2016.176 27811914

[34] Firestein GS. Evolving concepts of rheumatoid arthritis. Nature (2003) 423:356–61. 10.1038/nature01661 12748655

[35] Schmitt N Bentebibel SE Ueno H. Phenotype and functions of memory Tfh cells in human blood. Trends Immunol (2014) 35:436–42. 10.1016/j.it.2014.06.002 24998903 PMC4152409

[36] Tsai LM Yu D. Follicular helper T-cell memory: establishing new frontiers during antibody response. Immunol Cell Biol (2014) 92:57–63. 10.1038/icb.2013.68 24189164

[37] Rao DA Gurish MF Marshall JL Slowikowski K Fonseka CY Liu Y Pathologically expanded peripheral T helper cell subset drives B cells in rheumatoid arthritis. Nature (2017) 542:110–4. 10.1038/nature20810 28150777 PMC5349321

[38] Kleinsteuber K Heesch K Schattling S Sander-Juelch C Mock U Riecken K SOCS3 promotes interleukin-17 expression of human T cells. Blood (2012) 120:4374–82. 10.1182/blood-2011-11-392738 23033269

[39] Hillmer EJ Zhang H Li HS Watowich SS STAT3 signaling in immunity. Cytokine Growth Factor Rev (2016) 31:1–15. 10.1016/j.cytogfr.2016.05.001 27185365 PMC5050093

[40] Takeda K Clausen BE Kaisho T Tsujimura T Terada N Forster I Enhanced Th1 activity and development of chronic enterocolitis in mice devoid of Stat3 in macrophages and neutrophils. Immunity (1999) 10:39–49. 10.1016/S1074-7613(00)80005-9 10023769

[41] Yang XO Panopoulos AD Nurieva R Chang SH Wang D Watowich SS STAT3 regulates cytokine-mediated generation of inflammatory helper T cells. J Biol Chem (2007) 282:9358–63. 10.1074/jbc.C600321200 17277312

[42] Zhou L Ivanov II Spolski R Min R Shenderov K Egawa T IL-6 programs T(H)-17 cell differentiation by promoting sequential engagement of the IL-21 and IL-23 pathways. Nat Immunol (2007) 8:967–74. 10.1038/ni1488 17581537

[43] Stritesky GL Muthukrishnan R Sehra S Goswami R Pham D Travers J The transcription factor STAT3 is required for T helper 2 cell development. Immunity (2011) 34:39–49. 10.1016/j.immuni.2010.12.013 21215659 PMC3040244

[44] Liu R Wu Q Su D Che N Chen H Geng L A regulatory effect of IL-21 on T follicular helper-like cell and B cell in rheumatoid arthritis. Arthritis Res Ther (2012) 14:R255. 10.1186/ar4100 23176102 PMC3674600

[45] Misra DP Agarwal V Sharma A Wakhlu A Negi VS 2016 update of the EULAR recommendations for the management of rheumatoid arthritis: a Utopia beyond patients in low/middle income countries? Ann Rheum Dis (2017) 76:e47 10.1136/annrheumdis-2017-211446 28347992

[46] Saito S Suzuki K Yoshimoto K Kaneko Y Matsumoto Y Yamaoka K A new bioassay for measuring the strength of IL-6/STAT3 signal inhibition by tocilizumab in patients with rheumatoid arthritis. Arthritis Res Ther (2017) 19:231. 10.1186/s13075-017-1434-6 29041951 PMC5645925

[47] Chavele KM Merry E Ehrenstein MR. Cutting edge: circulating plasmablasts induce the differentiation of human T follicular helper cells via IL-6 production. J Immunol (2015) 194:2482–5. 10.4049/jimmunol.1401190 25681343 PMC4356730

[48] Aletaha D Neogi T Silman AJ Funovits J Felson DT Bingham CO III 2010 rheumatoid arthritis classification criteria: an American College of Rheumatology/European League against rheumatism collaborative initiative. Arthritis Rheum (2010) 62:2569–81. 10.1002/art.27584 20872595

