# Corrigendum: Signal Transducer and Activator of Transcription 3 Hyperactivation Associates With Follicular Helper T Cell Differentiation and Disease Activity in Rheumatoid Arthritis

**DOI:** 10.3389/fimmu.2019.02008

**Published:** 2019-08-22

**Authors:** Jun Deng, Chaofan Fan, Xin Gao, Qunxiong Zeng, Ruru Guo, Yunbo Wei, Zhian Chen, Yanan Chen, Dongcheng Gong, Jia Feng, Yan Xia, Shifei Xiang, Shushi Gong, Lin Yuan, Wei Shen, Wenyan Shen, Lin Lin, Ting Jiang, Dongyi He, Liangjing Lu, Xiaoxiang Chen, Di Yu

**Affiliations:** ^1^China-Australia Centre for Personalised Immunology, Renji Hospital, Shanghai Jiao Tong University School of Medicine, Shanghai, China; ^2^Department of Rheumatology, Shanghai Institute of Rheumatology, Renji Hospital, Shanghai Jiao Tong University School of Medicine, Shanghai, China; ^3^Hubei Provincial Key Laboratory of Occurrence and Intervention of Rheumatic Diseases, Affiliated Hospital of Hubei University for Nationalities, Enshi, China; ^4^Department of Immunology and Infectious Disease, John Curtin School of Medical Research, The Australian National University, Canberra, ACT, Australia; ^5^Laboratory of Immunology for Environment and Health, Shandong Analysis and Test Center, Qilu University of Technology, Shandong Academy of Sciences, Jinan, China; ^6^Department of Rheumatology, Affiliated Hospital of Hubei University for Nationalities, Enshi, China; ^7^Department of Laboratory Medicine, Renji Hospital, Shanghai Jiao Tong University School of Medicine, Shanghai, China; ^8^Department of Laboratory Medicine, Ruijin Hospital, Shanghai Jiao Tong University School of Medicine, Shanghai, China; ^9^Guanghua Hospital of Integrative Chinese and Western Medicine, Shanghai, China

**Keywords:** rheumatoid arthritis, patient, follicular helper T cells, signal transducer and activator of transcription 3, phosphorylation, IL-6

In the original article, there was a mistake in Figure 2 as published. One diagram (DAS28 vs. Th2), was mistakenly duplicated from another diagram (DAS28 vs. Th1) during the figure preparation. The corrected Figure 2 appears below.

**Figure 2:**
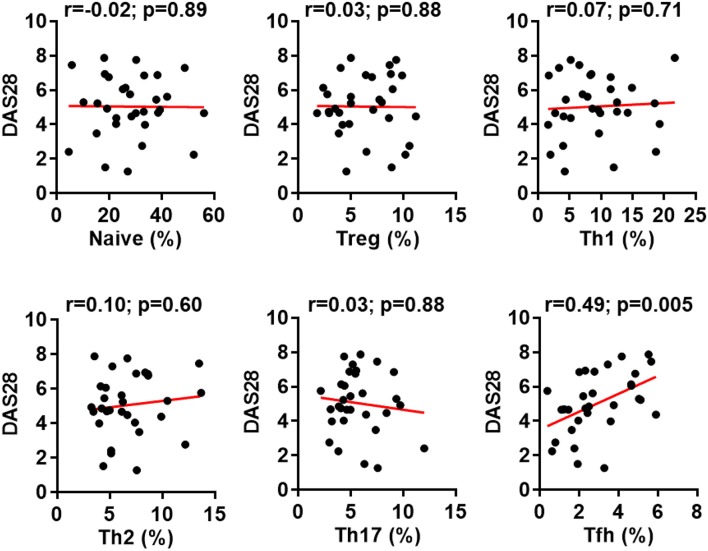
Increased follicular helper T (Tfh) cell differentiation correlates with rheumatoid arthritis (RA) disease activity. The percentages of CD4^+^ T cell subsets in the peripheral blood mononuclear cells from patients with RA were analyzed as Figure 1. The correlation between the frequencies of these subsets and the disease activities measured by DAS28 were determined using Spearman's correlation coefficient.

The authors apologize for this error and state that this does not change the scientific conclusions of the article in any way. The original article has been updated.

