# Supplementary material for: Signal Transducer and Activator of Transcription 3 Hyperactivation Associates With Follicular Helper T Cell Differentiation and Disease Activity in Rheumatoid Arthritis
Source: Front Immunol. 2018 Jun 4;9:1226. doi: 10.3389/fimmu.2018.01226 (PMC5994589; doi:10.3389/fimmu.2018.01226)
Supplement: Supplementary file 1 [file data_sheet_1.PDF]

STAT3 hyperactivation associates with follicular helper T cell differentiation and disease activity in rheumatoid arthritis

Supplementary data

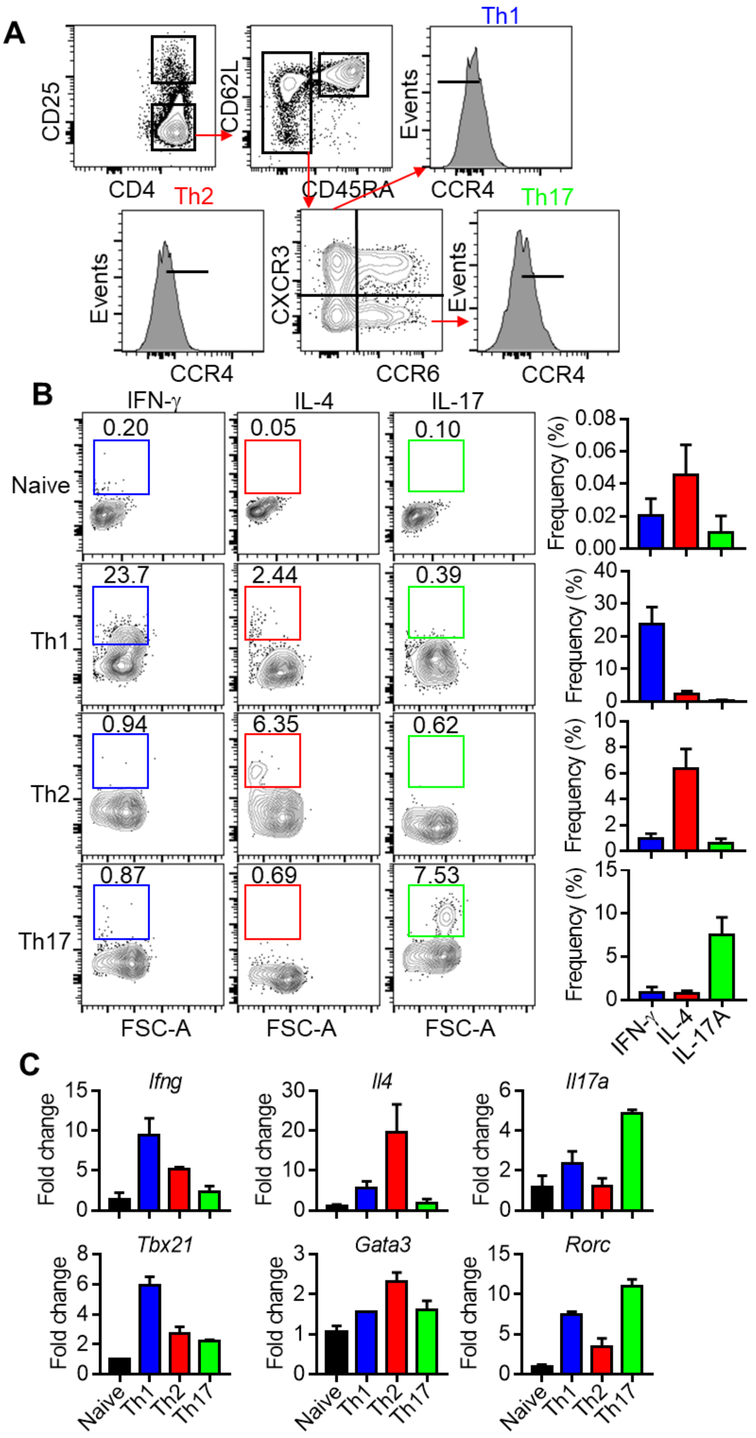

Figure S1. **CD4$^+$ T cell subsets sorting and intracellular cytokines staining.**

(A) FACS gating strategies of naïve (CD25$^-$CD45RA$^+$CD62L$^+$), Th1 (CD25$^-$CD45RA$^-$CXCR3$^+$CCR6$^-$CCR4$^-$), Th2 (CD25$^-$CD45RA$^-$CXCR3$^-$CCR6$^-$CCR4$^+$), Th17 (CD25$^-$CD45RA$^-$CXCR3$^-$CCR6$^+$CCR4$^+$), after the removal of 7ADD$^+$ died cells from the PBMC of healthy controls.

(B) FACS assay and statistics of IFN-γ, IL-4 and IL-17 in each subset. Cells were stimulated with PMA, Ionomycin and brefeldin A for 4 hours. IFN-γ, IL-4 and IL-17 were intracellularly stained.

(C). mRNA expression levels of and *Ifng*, *Il4*, *Il17a*, *Tbx21*, *Gata3* and *Rorc* were measured by q-PCR.

Data obtained healthy controls (n=5) were shown mean±SEM

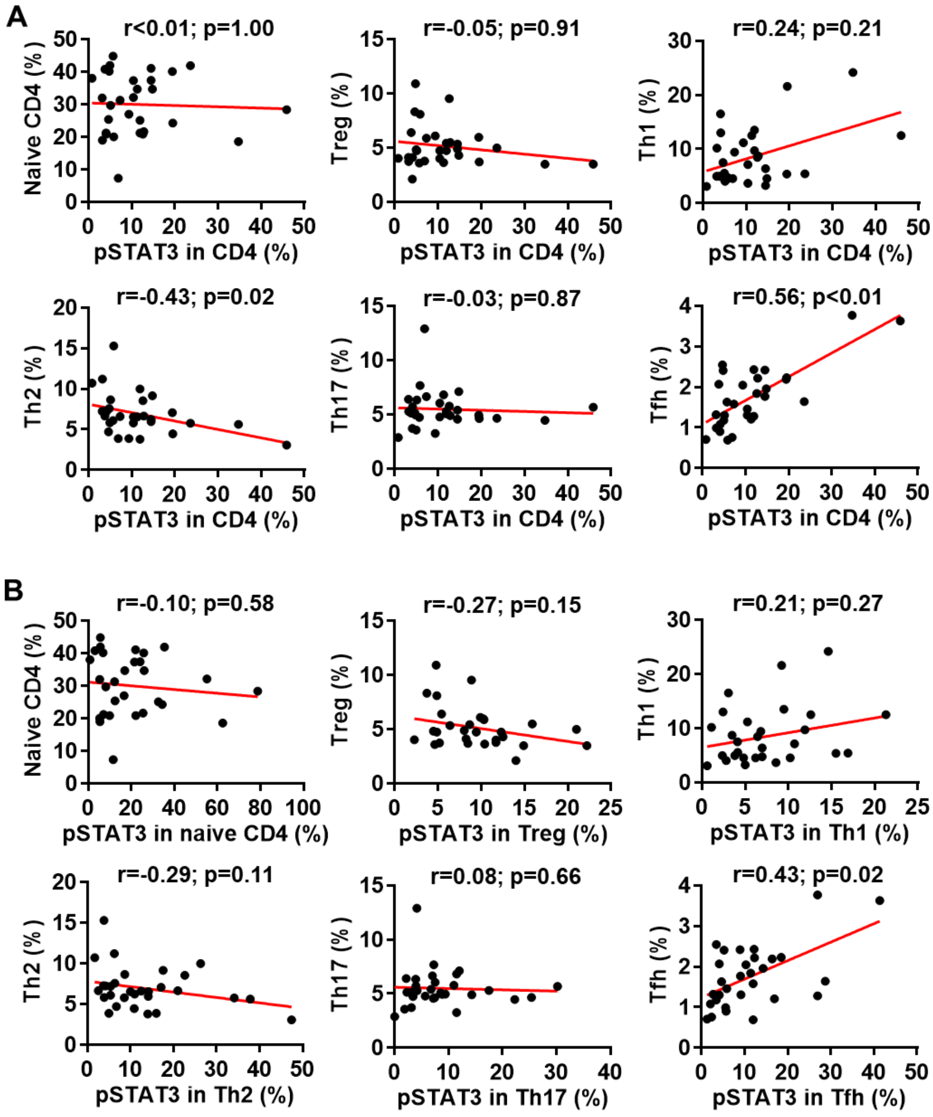

Figure S2. STATS3 hyperactivation correlates with aberrant Tfh differentiation in patients with RA.

(A, B) Statistics showing the relationship between the frequencies of indicated $CD4^{+}$ T cell subsets with the pSTAT3 expression in total $CD4^{+}$ T cells (**A**) or in each individual subsets (**B**) in the PBMCs from healthy controls (n=30). The correlation was determined using Spearman's correlation coefficient.
